# Examining multilevel resilience as a moderator of associations between gendered racial microaggressions and postpartum mental health and functioning

**DOI:** 10.1016/j.jpsychires.2026.04.002

**Published:** 2026-04-08

**Authors:** Frances M. Howell, Natalie Boychuk, Latima Collins, Melissa J. Dupont-Reyes, Sarah Nowlin, Lisa Levine, Elizabeth A. Howell, Teresa Janevic

**Affiliations:** a Department of Epidemiology, Columbia University Mailman School of Public Health, United States; b Department of Obstetrics and Gynecology, University of Pennsylvania, United States; c Department of Sociomedical Sciences, Columbia University Mailman School of Public Health, United States; d Icahn School of Medicine at Mount Sinai, Department of Population Health Science and Policy, United States; e Mount Sinai Health System, Center for Nursing Research and Innovation, United States

**Keywords:** Gendered racial microaggressions, Global majority birthing people, Postpartum depression, Postpartum functioning, Resilience

## Abstract

The United States is currently facing a maternal health crisis, and mental health issues are a leading preventable cause of maternal mortality. We assessed the influence of resilience on the relationship between gendered racial microaggressions (GRM) and postpartum depressive symptoms and functioning. We analyzed data among 265 postpartum Global Majority participants across four Philadelphia and New York hospitals. We administered the adapted Gendered Racial Microaggressions Scale (α = 0.97) to participants before discharge. Postpartum depressive symptoms, postpartum functioning, and resilience were assessed at 3 months postpartum using the Edinburgh Postnatal Depression Scale, the Patient-Reported Outcomes Measurement Information System Global Short Form, the Collective Efficacy Scale, and the Connor-Davidson Resilience Scale, respectively. Multilinear regressions tested the associations between GRM and postpartum depressive symptoms and functioning. Moderation analysis examined how community and personal resilience changed the relationship between GRM and postpartum depressive symptoms and functioning. Experiencing GRM during obstetric care was associated with postpartum depressive symptoms and lower postpartum functioning symptoms. Personal resilience moderated the association between GRM and postpartum functioning; this moderation was not observed for postpartum depressive symptoms. Community resilience did not moderate the association between GRM or postpartum depression and functioning symptoms. Gendered racial microaggressions, in obstetric contexts, harm postpartum mental and physical health. However, personal resilience may mitigate the impact of racism in obstetric care on postpartum health.

## 1. Introduction

Currently, the United States (US) is facing a maternal health crisis characterized by inequities among Global Majority^1^ birthing people during pregnancy, birth, and the postpartum period. In 2021, the maternal mortality rate for Black birthing people was 2.6 times the rate for White birthing people (Hoyert, 2023), and severe maternal morbidity is 1.3 and 1.2 times higher among Asian and Latinx populations, respectively, than their White counterparts (Howell, 2018). Notably, mental health conditions have been identified as a leading underlying cause of preventable pregnancy-related mortality (Gimbel et al., 2024). Increasing evidence has emerged revealing significant racial and ethnic disparities in postpartum mental health outcomes: Global Majority women are more likely to experience postpartum depression, while Black women are less likely to receive postpartum mental health treatment compared to other racial and ethnic groups (Njoroge et al., 2022). Even after adjusting for factors such as demographics, physical symptoms, self-efficacy, and social trust, the risk of postpartum depression among Black and Hispanic women remains elevated (Howell et al., 2005). Research suggests that other factors may be driving postpartum mental health-related mortality and inequities.

A significant factor related to mental and obstetric health outcome inequities is systemic racism, which is deeply embedded in reproductive medicine. Its insidious influences can be traced back to early obstetrics during slavery, in which Black women’s reproduction was exploited and commodified (Davis, 2019). Recent research has underscored the continued impact of obstetric racism on postpartum depression and anxiety, highlighting that experiences of distress due to racism in the year before birth are associated with an increased prevalence of postpartum depression (Bossick et al., 2022). The undercurrent of racism in the obstetric health system plays out in a myriad of ways, from the acute and deadly to the more subtle and everyday experiences of gendered racial microaggressions (GRM). Obstetric racism can manifest as GRM, an insidious and subtle form of everyday experiences of gendered and racial discrimination, such as being neglected in health care settings (Lewis and Neville, 2015). In the context of obstetric medical settings, research has shown that gendered racial microaggressions are experienced as manifestations of feeling neglected and poor quality of care, feeling excluded from healthcare resources and medical advice, and poor perinatal outcomes among Asian, Black, and Hispanic pregnant people (Crawford et al., 2022; Davis, 2019). Existing research has also demonstrated the adverse impacts of GRM on mental and physical health among postpartum Global Majority populations. For instance, a recent study revealed that experiencing GRM during pregnancy and birth is associated with poorer postpartum blood pressure trajectories (Janevic et al., 2025). Likewise, research highlights that among Black women, GRM is associated with increased psychological distress, traumatic stress symptoms, anxiety, and depression (Moody and Lewis, 2019). Given that Global Majority pregnant and birthing people are more likely to give birth in a hospital setting (Howell et al., 2018), the quality of clinical care that they receive during pregnancy represents a vital determinant of postpartum mental health.

As noted, the fourth trimester – up to one year postpartum – is a significant period marked by health inequities that contribute to the US maternal health crisis. During the fourth trimester, experiencing discrimination and bias during pregnancy and birth is associated with lower postpartum visit attendance (Scott & Davis, 2022). Postpartum individuals have reported a lack of clear guidance on postpartum recovery, and postpartum care systems are lacking (Scott & Davis, 2022). Furthermore, postpartum functioning is associated with factors related to birth experiences, such as parity and type of delivery, and evidence suggests that postpartum functioning—the physical and psychological recovery and well-being of an individual after giving birth—is associated with postpartum mental health outcomes (Barkin et al., 2017). Taken together, experiencing discrimination during pregnancy and childbirth can impact postpartum functioning, which, in turn, can lead to poor postpartum mental health outcomes.

Despite the detrimental influence of experiencing GRM on postpartum mental health and functioning, resilience may have a mitigating effect by enabling postpartum people to adapt to their circumstances and reduce stress. Research suggests that personal resilience plays a role in buffering traumatic stress among Black women who frequently experience GRM (Watson and Henderson, 2023). Higher levels of personal resilience, such as having social support and mindfulness, during the perinatal and postpartum periods, are associated with a reduction in postpartum mental disorders among Global Majority birthing people (Hajure et al., 2024). Furthermore, the social environment and community that people live in factor into community-level resilience - social connection, collective action, and cohesion among neighbors or within a community (Sampson et al., 1997). Comfortable social environments that include well-maintained housing, quality grocery stores, parks, and social cohesion can create community resilience that may buffer chronic stressors and poor health outcomes (Mullings and Wali, 2001). Therefore, just as resilience buffers against postpartum depression, community and personal resilience might be a protective factor of postpartum functioning among Global Majority of birthing people who experience GRM during pregnancy and birth.

There is a paucity of literature regarding how experiences of GRM during pregnancy and birth influence postpartum functioning and mental health outcomes and how the role of resilience may buffer these effects. Research on postpartum health outcomes tends to exclude racially and ethnically diverse populations of birthing people who have a higher likelihood of experiencing GRM and its disparate impact on postpartum mental health and functioning outcomes. Thus, this study aims to assess community and personal resilience as a moderator of the relationship between experiences of GRM in obstetric contexts and postpartum functioning and depression. Our hypotheses are threefold. We hypothesize that: a) experiencing GRM in obstetric contexts is associated with higher rates of postpartum depressive symptoms and lower postpartum functioning symptoms; b) community and personal resilience moderate the relationship between experiencing GRM and higher scores of postpartum depressive symptoms; and c) community and personal resilience moderate the relationship between experiences of GRM and lower postpartum functioning symptoms.

## 2. Methods

### 2.1. Procedure

This study is a secondary data analysis of the VIBE Study (Howell et al., 2024; Janevic et al., 2025). Our multi-site prospective postpartum cohort study was conducted between 2022 and 2023 across four hospitals in New York City and Philadelphia. Eligibility included self-identifying as Asian, Black, or Hispanic; speaking English or Spanish; and having the ability to text message, as participants would be required to send and receive text messages to complete the study procedures as outlined in Howell et. al (2024). Patients in the postpartum unit at each hospital were invited to participate in the study and enrolled prior to discharge. Research coordinators obtained informed consent and administered a baseline survey to enrolled participants, hosted on Research Electronic Data Capture (REDCap; Harris et al., 2019), at the participant’s bedside in the postpartum unit before being discharged. At the end of the three-month enrollment period, participants completed follow-up surveys. The VIBE study obtained IRB approval. For further details regarding participant recruitment and enrollment, please refer to the previously published manuscripts, Author, Blinded for Review, 2024; Authors, Blinded for Review, 2025.

### 2.2. Participants

Data was analyzed from n = 265 postpartum people. Participants were 31 years old on average (*SD*, 6.13), and identified as Hispanic (41.1%), Black (39.2%), ‘More than one race/Other’ (10.6%), and Asian (9.1%). Most reported being insured by public health insurance (48.4%), delivered vaginally (69.1%), were multiparous (65.3%), had no previous therapy (89.1%), and were in a relationship (75.5%). For additional sample characteristics, see Table 1.

### 2.3. Materials

Gendered racial microaggressions were assessed using an adapted version of the Gendered Racial Microaggressions Scale in English and Spanish (GRMS; Lewis and Neville, 2015). GRMS is a scale that measures the frequency and stress appraisal of gendered microaggressions experienced by Black women specifically (Lewis and Neville, 2015). To adapt the GRMS scale to capture instances of gendered racial microaggressions in obstetric contexts (i.e., interpersonal experiences of racism in obstetric clinical settings during pregnancy and birth) to a multiethnic and bilingual cohort, the research team consulted their advisory board comprised of Global Majority women who work with communities similar to our participant population (in positions such as doulas, OB/GYNS, and birth justice advocates) on which items to choose and how to change the language on said items to reflect their first-hand knowledge of racism in obstetric contexts (Howell et al., 2024). The research study team then extensively reviewed the literature on Asian, Black, and Latina women’s experiences with gendered racial microaggression, both more broadly and in the context of maternal healthcare, to ensure that the adapted nine items reflected the existing research as it pertains to these racial and ethnic groups. Based on feedback from the study’s advisory board’s experience working with pregnant Global Majority individuals, as well as the extensive literature on Global Majority women’s experiences with racism, gendered racism, and gendered racial microaggressions during pregnancy, birth, and postpartum, the researchers selected a total of 9-items from Factor B: Silence and Marginalization and Factor D; Angry Black Women Stereotypes of the original scale (Howell et al., 2024). Following the prompt of “thinking about over the course of your pregnancy care and delivery care in the hospital, rate the following.” Participants responded on a scale from 0 (never) to 5 (all the time) to items such as “I have felt unheard” and “Someone has tried to ‘put me in my place’,” please refer to the online supplementary materials for the adapted nine-item scale. The adapted nine-item GRMS (α = 0.89) is valid for use among Global Majority pregnant and birthing people who speak English and Spanish (blinded for review). Further details on the development and validation of our nine-item adapted GRMS scale are provided in (Howell et al., 2024).

Postpartum depression was assessed using the Edinburgh Postnatal Depression Scale (EPDS; Cox et al., 1987). The EPDS is a 10-item scale (α = 0.87), with questions such as “I have felt sad or miserable,” rated on a scale from 0 to 3 with a recommended total score of 13 to screen for postpartum depression. EPDS is validated (α = 0.86) among Asian, Black, and Hispanic Global Majority birthing people (Shakeel et al., 2018).

Postpartum functioning was measured using the Patient-Reported Outcomes Measurement Information System Global Short Form (PROMIS). PROMIS is a validated 10-item scale capturing physical and mental health functioning and includes items such as “In general, would you say your health is: excellent, very good, good, fair, or poor.” Nine items are on a Likert-type scale from 1 to 5, and one item measures pain severity on a scale from 1 to 10. PROMIS (α = 0.89) has been assessed for its validity across diverse Global majority clinical populations and among postpartum populations (Lundsberg et al., 2018). Lower scores indicate poorer functioning.

Resilience was measured at the community and personal level using the social cohesion and trust subscale from the Collective Efficacy Scale (CES; Sampson et al., 1997) and the 10-item Connor-Davidson Resilience Scale (CD-RISC10; Connor and Davidson, 2003). The CES social cohesion and trust subscale includes five items, such as “People in this neighborhood can be trusted,” rated on a 5-point scale from 1 to 5, and has been used to assess the association between neighborhoods and physical and mental health (McCloskey and Maguire-Jack, 2021). The CD-RISC10 (α = 0.89) assesses personal resilience using five response options, ranging from 0 (never) to 4 (almost always), on items such as “I am able to adapt when changes occur.” The CD-RISC10 has been used to assess protective factors against postpartum stress (Mollard et al., 2021). Higher scores for both measures indicate more resilience.

Participants’ self-reported demographic information and the nine-item adapted GRMS scale were collected via a survey administered at baseline (prior to discharge). We obtained participants’ delivery mode (i.e., vaginal or cesarean) and parity from their electronic medical records. The three-month follow-up survey captured whether participants received therapy or mental health counseling within a year before pregnancy, along with their postpartum depressive symptoms (which was obtained at both baseline and follow-up), postpartum functioning scores, and community and personal resilience scores. We operationalized scores on the GRMS, postpartum depressive symptoms, and postpartum functioning measures as continuous in our analyses.

### 2.4. Data analysis

Data were analyzed using R (v4.4.0). Descriptive statistics were used to analyze participant sociodemographic characteristics. Pearson r correlations were used to examine the relationships between GRMS, postpartum depressive symptoms, and postpartum functioning. A logarithmic transformation was applied because the distributions of GRMS, postpartum depressive symptoms, and postpartum functioning were skewed, and our findings are based on these transformed data. Next, we estimated four linear regression models, unadjusted and controlling for race, delivery mode, parity, relationship status, and previous therapy, to examine the direct relationship between (i) GRMS and postpartum depressive symptoms and (ii) GRMS and postpartum functioning.

We then conducted moderation analyses to examine whether personal or community-level resilience moderates the relationship between (i) GRMS and postpartum depressive symptoms and (ii) GRMS and postpartum functioning. For each outcome, GRMS was entered as the predictor, and the interaction of community resilience with GRMS was entered as a moderator. Models were repeated with an interaction term between personal resilience and GRMS. Lastly, we conducted a post hoc sensitivity analysis to explore the relationship between both baseline and follow-up EPDS scores and GRMS.

## 3. Results

### 3.1. Bivariate analyses

Pearson correlations were computed to assess the relationships between gendered racial microaggressions (GRMS), postpartum depressive symptoms, and postpartum functioning. As reported in Table 2, there was a positive association between GRMS and postpartum depressive symptoms (*r* = 0.21), a negative relationship between GRMS and postpartum functioning (*r* = −0.13), and a negative relationship between postpartum depressive symptoms and postpartum functioning (*r* = −0.39).

### 3.2. Multivariate analyses

Adjusted linear regression analyses demonstrated that experiencing gendered racial microaggressions (GRM) was associated with higher follow-up postpartum depressive symptoms scores (*B*=0.25, *SE* = 0.05, *p* < 0.001). Similarly, sensitivity analyses adding baseline postpartum depressive scores as a covariate in the model revealed that GRM was associated with postpartum depressive symptoms (*B*=0.17, *SE* = 0.05, *p* < 0.01). GRM was also associated with lower postpartum functioning scores (*B* = −0.04, *SE* = 0.01, *p* < 0.001), as shown in Tables 3-4.

We then examined whether community or personal resilience moderates the relationship between GRM and postpartum depressive symptoms, controlling for race and ethnicity, delivery mode, therapy, and relationship status. Results from these moderation models revealed that GRM was positively associated with postpartum depressive symptoms (*B*=0.18, *SE* = 0.06, p < 0.01), whereas community resilience was negatively associated with postpartum depressive symptoms (*B* = −*0.59, SE* = *0.06, p < 0.001)*. However, the interaction between GRM and community resilience did not moderate the association between GRM and postpartum depressive symptoms (see Table 5).

In moderation models that included personal resilience, GRM remained positively associated with postpartum depressive symptoms (B = 0.17, SE = 0.7, p < 0.01), whereas personal resilience was not significantly associated with postpartum depressive symptoms. The interaction between GRM and personal resilience, however, was not significant, indicating that it does not moderate the relationship between GRM and postpartum depressive symptoms (Table 6).

Next, we examined whether community or personal resilience moderates the relationship between GRM and postpartum functioning, adjusting for race and ethnicity, delivery mode, therapy, and relationship status. GRM was negatively associated with postpartum functioning (*B* = −*0.03, SE* = *0.01, p < 0.05*). Conversely, community resilience was positively associated with postpartum functioning *(B* = *0.08, SE* = *0.03, p <* 0.05). However, the interaction between GRM and community resilience was not significant, indicating that community resilience did not significantly moderate the relationship between GRM and postpartum functioning (Table 7). In our final model examining personal resilience as a moderator of the relationship between GRM and postpartum functioning, GRM did not significantly predict postpartum functioning. However, the interaction between GRM and personal resilience was significant (*B* = −*0.06, SE* = *0.02, p < 0.01*). This interaction suggests that higher levels of personal resilience scores mitigate the negative association between GRM and postpartum functioning (Table 8).

## 4. Discussion

Corresponding to our three hypotheses, key findings from the current secondary data analysis are: (1) experiencing gendered racial microaggressions (GRM) during obstetric care were positively associated with postpartum depressive symptoms and negatively associated with post-partum functioning; (2) community and personal resilience does not moderate the relationship between experiencing GRM and postpartum depressive symptoms; and (3) personal resilience modified the relationship between experiencing GRM and postpartum functioning, whereas community resilience did not.

Our findings of GRM's association with higher postpartum depressive symptoms and lower postpartum functioning are consistent with previous research on these topics. For instance, research demonstrates that Global Majority birthing people experience differential treatment and disrespect during patient-provider prenatal care interactions, which may be experienced as obstetric racism and gendered racial microaggressions (Floyd James et al., 2024; Janevic et al., 2020; McLemore et al., 2018). Research examining postpartum depressive symptoms suggests that symptoms of postpartum depression are higher among Global Majority of birthing people and associated with decreased postpartum functioning (Barkin et al., 2017; Howell et al., 2005). Our results extend this research by illustrating that experiences of racism during obstetric care play a significant part in the decreased postpartum functioning and poorer mental health experienced by the Global Majority in the postpartum period.

We found that personal resilience moderates associations between gendered racial microaggressions, and postpartum functioning. This finding is consistent with reports of gender racial microaggression's associations with psychological distress and adverse physical health among Black women (Lewis et al., 2017). Resilience is also associated with symptoms of postpartum depression. Research has demonstrated that resilience moderated the relationship between childhood trauma and postpartum depression, suggesting that, overall, resilience plays a vital role in postpartum mental health (Baattaiah et al., 2023; Sexton et al., 2015). Conversely, a qualitative study on maternal resilience among low-income and minoritized women suggests that even though interpersonal and community support systems may contribute to maternal resilience, they may also be a source of stress and negative social support (Wakeel et al., 2025). More research is needed to understand better non-Black Global Majority postpartum people's resiliency and protective strategies against gendered racial microaggressions in obstetric contexts. Our results not only build on previous research exploring the role that racism and gendered racial microaggressions have on negatively impacting both mental and physical outcomes among postpartum Global Majority populations but also reveals the role and limitations of community and personal resilience in moderating the impact of racism on postpartum outcomes.

### 4.1. Strengths and limitations

Our study has several strengths and limitations worth noting. First, we did not have access to a diagnosis of perinatal depression, other mood disorders, or past experiences with postpartum depression and other mental health conditions. However, we did adjust for self-reported previous therapy or mental health counseling within the past year in our models (see Tables 3-8), and ran a sensitivity analysis that includes baseline depressive symptom scores (see Table 3) to understand the relationship postpartum depressive symptoms and experiencing gendered racial microaggressions. Given that a history of prior mood disorders and prenatal depression predicts postpartum depression (Beck, 1996), previous mental health struggles not captured in the study, unreported mood disorder diagnoses, and the possibility of systemic obstacles to accessing mental health services, we cannot fully rule out the possibility of pre-pregnancy and early postpartum (12-weeks post birth) depressive symptoms effecting our results. Although this is a limitation for the analysis of the main effect of gendered racial microaggression on postpartum depressive symptoms, the presence of prior depression and depressive symptoms would not change our conclusions that neither personal nor community resilience mediates this relationship. Furthermore, the exclusive focus on GRM in obstetric contexts does not account for broader experiences of racism that may influence participants’ postpartum mental health and functioning. Nonetheless, our investigation of experiences of GRM in obstetric contexts on postpartum mental health and functioning is a strength since our results reveal that more subtle and covert forms of racism contribute to racial and ethnic inequities in adverse psychological and physical postpartum outcomes.

Additionally, due to a lack of power and small sample sizes among some racial and ethnic subgroups, we were unable to conduct further analyses exploring the impact of gendered racial microaggressions, multilevel resilience, and mental and physical health, specific to race and ethnicity. Furthermore, However, most research on the association of racism and postpartum mental and physical health focuses on either a single race and ethnicity or compares Global Majority postpartum populations to those of White postpartum people. Our study uniquely comprises a diverse sample of Asian, Black, and Latina postpartum individuals who spoke both English and Spanish. Therefore, we cannot ascertain how gendered racial microaggressions in obstetric contexts may impact Global Majority subgroups more broadly.

Second, we acknowledge recent criticisms of the limitations of quantifying both experiences of gendered racial microaggressions, which may be viewed as subjective instances of “bad care” rather than a form of racism experienced during pregnancy and birth. In particular, we acknowledge that the study team's psychometric evaluation of the adapted 9-item GRMS scale did not include test-retest reliability, IRT analysis demonstrated that “the unidimensional model showed some misspecification through residual correlation p.7,” and that our analyses revealed “observed floor effects in the GRMS not reported in the construction and validation of the original GRMS p.14” (Howell et al., 2024). Regardless these limitations do not diminish the validity of our adapted 9-item scale and psychometric support that our validation study provided for a scale that captures subtle forms of racism in obstetric contexts (Howell et al., 2024).

Another recent criticism brought forth by public health and mental health researchers underscores the limitations of quantifying resilience to understand how individuals respond to and cope with varying forms of distress and adverse events. Moreover, despite previous research demonstrating community and personal resilience's role in mitigating the impacts of adverse birth outcomes and limiting exposure to inter-personal racism, health equity scholars point out that a resilience framework “places the locus of intervention for structural harms within the marginalized individual” and not the structures that cause harm (p. 340, Suslovic and Lett, 2024); and fail to recognize how resilience may be a short-term solution to coping with distress with long-term negative health consequences (e.g., Sojourner Syndrome; Mullings, 2005). Put differently, there should be a focus on advocating for structural changes, such as policies at the federal level (i.e., midwifery and doula care reimbursement programs and legislation that supports paid parental leave, affordable childcare, and child tax credit) and institutional level (i.e., mandatory written postpartum recovery plans given at discharge) instead of interventions focusing on resilience-only framework to address inequities. Future research should focus on both the efficacy of interventions to reduce the occurrence of gendered racial microaggressions and provide postpartum patients with the tools to cope with the physical and mental transition to parenthood that may impact their postpartum functioning and mental health.

## 5. Conclusions

This secondary analysis of VIBE study data from a multi-site postpartum cohort study advances our understanding of how gendered racial microaggressions experienced in obstetric contexts are associated with symptoms of postpartum depression and functioning and highlights the role of community and personal resilience in mitigating these associations. Our findings align with supporting research that highlights how subtle forms of racism, such as gendered racial microaggressions, that may be easily dismissed in obstetric clinical settings have adverse effects on postpartum mental and physical health and provide further insight into how racism contributes to racial and ethnic maternal health inequities in the US. Future research should continue to investigate structural, neighborhood and built environment factors, social, and interpersonal causes of racism and GRM in obstetric contexts and create more holistic approaches, such as integrating mental health in prenatal care, to reduce the occurrence of postpartum depression and negative postpartum physical functioning.

## Figures and Tables

**Table 1:** Participant sociodemographic characteristics.

Characteristic	N (%)	M	SD
Age		31	6.13
Race			
Asian	23 (9.1%)		
Black/African American	104 (39.2%)		
Hispanic/Latino	109 (41.1%)		
More than one race/Other	28 (10.6%)		
Nativity			
US-Born	123 (46.4%)		
Foreign-Born	142 (53.6%)		
Parity			
Nulliparous (no prior births)	92 (34.7%)		
Multiparous (at least 1 prior birth)	173 (65.3%)		
Delivery Method			
Vaginal	183 (69.1%)		
Cesarean	82 (30.9%)		
State			
PA	144 (54.3%)		
NY	121 (45.7%)		
Education			
Less than HS	45 (17%)		
HS or GED	86 (32.5%)		
Some College	63 (23.8%)		
BA or Higher	70 (26.4%)		
Don't Know/Prefer not to answer	1 (0.4%)		
Relationship Status			
Single	63 (23.8%)		
Married, living with a partner, or in a relationship	200 (75.5%)		
Divorced/Separated	1 (0.4%)		
Widow	1 (0.4%)		
Therapy before pregnancy			
Yes	29 (10.9%)		
No	236 (89.1%)		
Community Resilience			
High	128 (48.3%)		
Low	137 (51.7%)		
Individual Resilience			
High	135 (50.9%)		
Low	130 (49.1%)		

**Table 2:** Pearson correlations among gendered racial microaggressions, postpartum depression, and postpartum functioning.

	*n*	*M*	*SD*	1	2	3	4
1. GRMS	265	2.87	6.38	-			
. EPDS^a^	265	5.12	4.26	0.22**			
3. EPDS^b^	265	4.44	4.00	0.21**	0.52***	-	−0.30
4. PROMIS	265	51.28	8.33	−0.13*	−0.28	−0.39**	-

*Note.* GRM = Gendered Racial Microaggressions, EDPS = Postpartum Depressive Symptoms, PROMIS = Postpartum Functioning.

** *p* < 0.001

** *p* < 0.01

* *p* < 0.05.

a EPDS baseline score.

b EPDS 3-month follow-up score.

**Table 3:** Unadjusted and adjusted regression analyses for GRM predicting postpartum depressive symptoms.

	Postpartum Depressive Symptoms (EDPS)^a^								
	Model 1			Model 2			Model 3		
	*B*	*SE*	95%CI	*B*	*SE*	95% CI	*B*	*SE*	95% CI
GRM^a^	0.24**	0.05	0.15, 0.35	0.25**	0.05	0.15, 0.35	0.17**	0.05	0.06, 0.24
EPDS at Baseline							0.48***	0.06	0.38, 0.61
Asian				0.21	0.28	−0.77, 0.34	−0.05	0.25	−0.71, 0.26
Hispanic/Latina				−0.08	0.17	−0.41, 0.25	−0.06	0.15	−0.43, 0.15
Other				−0.62*	0.26	−1.34, −0.11	−0.12*	0.23	−0.95, −0.03
Delivery				−0.01	0.16	−033, 0.30)	−0.07	0.14	−0.47, 0.08
Parity				−0.02	0.16	−0.35, 0.27	−0.052	0.14	−0.4, 0.14
Single Relationship Status				0.16*	0.18	0.11, 0.82	0.07	0.16	−0.11, 0.53
Previous Therapy				0.06	0.24	−0.19, 0.75	0.05	0.21	
*R* ^*2*^	0.08			0.13			0.33		
*Adjusted R* ^*2*^	0.08			0.10			0.03		
*F*	23.89			4.71			13.41		

*Note.* GRM = Gendered Racial Microaggressions, EPDS = Postpartum Depressive Symptoms.

Model 1 = Unadjusted Linear Regression Model, Overall Model 1 Statistics: *F* (1, 270) = 23.89, *p* < 0.001, *R*^*2*^ = 0.08.

Model 2 = Adjusted Linear Regression Model, Overall Model 2 Statistics: *F* (8, 250) = 4.71, *p* < 0.001, *R*^*2*^ = 0.13.

Model 3 = Adjusted Linear Regression for Sensitivity Analysis, Overall Model 3 Statistics: *F* (9, 249) = 13.41, *p* < 0.001, *R*2 = 0.33

a Logarithmic transformed variable.

** *p* < 0.001

* *p* < 0.01.

**Table 4:** Unadjusted and adjusted regression analyses for GRM predicting postpartum functioning.

	Postpartum Functioning (PROMIS)^a^					
	Model 1			Model 2		
	*B*	*SE*	95%CI	*B*	*SE*	95% CI
GRM^a^	−0.04***	0.01	−0.06, −0.02	−0.04***	0.01	−0.06, −0.02
Asian				0.02	0.05	−0.09, 0.12
Hispanic/Latina				−0.5	0.03	−0.11, 0.02
Other				0.05	0.05	−0.05, 0.15
Delivery				0.02	0.03	−0.04, 0.08
Parity				−0.06	0.03	−0.12, 0.00
Single Relationship Status				−0.04	0.04	−0.11, 0.03
Previous Therapy				−0.02	0.05	−0.11, 0.07
*R* ^*2*^	0.07			0.11		
*Adjusted R* ^*2*^	0.07			0.08		
*F*	19.39			3.78		

*Note.* GRM = Gendered Racial Microaggressions, PROMIS = Postpartum Functioning.

Model 1 = Unadjusted Linear Regression Model, Overall Model 1 Statistics: F (1, 257) = 19.39, p < 0.001, R^2^ = 0.07 Model 2 = Adjusted Linear Regression Model, Overall Model 1 Statistics: F (8, 250) = 3.78, p < 0.001, R^2^ = 0.11

a Logarithmic transformed variable.

*** *p* < 0.001.

**Table 5:** Unadjusted and adjusted analysis for community resilience moderating GRM from postpartum depressive symptoms.

	Postpartum Depressive Symptoms (EPDS)^a^									
	Model 1					Model 2				
	*B*	*SE*	*t*	^*p*^	95% CI	*B*	*SE*	*t*	^*p*^	95% CI
GRM^a^	0.17	0.06	2.76	0.01*	0.05, 0.29	0.18	0.06	2.78	0.01*	0.05, 0.30
CR^a^	−0.59	0.17	−3.43	0.001**	−0.94, −0.25	−0.58	0.18	−3.09	0.001**	−0.93, −0.24
GRMS X CR	0.12	0.11	1.15	0.25	−0.09, 0.33	1.13	0.08	1.55	0.28	−0.09, 0.32
Asian						−0.19	0.28	−0.70	0.48	−0.74, 0.35
Hispanic/Latina						−0.02	0.17	−0.13	0.90	−0.35, 0.31
Other Race						−0.49	0.26	−1.91	0.06	−0.99, 0.02
Delivery Mode						0.01	0.16	0.06	0.95	−0.30, 0.32
Parity						−0.02	0.07	−0.25	0.80	−0.15, 0.11
Single Relationship Status						0.46	0.16	2.78	0.009**	0.11, 0.77
Previous Therapy						0.25	0.24	1.10	0.29	−0.21, 0.72
*R* ^*2*^	0.12					0.16				
*Adjusted R* ^*2*^	0.11					0.13				
*F*	12.41					4.99				

*Note.* EDPS = Postpartum Depressive Symptoms, GRM = Gendered Racial Microaggressions, CR = Community Resilience.

Model 1 = Unadjusted Linear Regression Model, Overall Model 1 Statistics: F (3, 255) = 12.41, p < 0.001, R^2^ = 0.12.

Model 2 = Adjusted Effect Modification Model, Overall Model 2 Statistics: F (8, 250) = 4.99, p < 0.001, R^2^ = 0.16

a Logarithmic transformed variable.

** p < 0.001

* p < 0.01.

**Table 6:** *Unadjusted and Adjusted Analysis for Personal Resilience Moderating GRM from* postpartum depressive symptoms.

	Postpartum Depressive Symptoms (EDPS)^a^									
	Model 1					Model 2				
	*B*	*SE*	*t*	^*p*^	95% CI	*B*	*SE*	*t*	^*p*^	95% CI
GRM^a^	0.17	0.07	2.53	0.01*	0.04, 0.29	0.17	0.07	2.66	0.008**	0.04, 0.30
PR^a^	−0.27	0.18	−1.53	0.13	−0.62, 0.08	−0.24	0.18	−1.34	0.18	−0.58, 0.11
GRM X PR	0.20	0.10	1.90	0.06	−0.01, 0.41	0.19	0.11	1.80	0.07	−0.02, 0.40
Asian						−0.26	0.28	−0.91	0.37	−0.82, 0.30
Hispanic/Latina						−0.09	0.17	−0.51	0.61	−0.24, 0.25
Other Race						−0.61	0.26	−2.33	0.02*	−1.12, −0.09
Delivery Mode						−0.03	0.16	−0.16	0.87	−0.33, 0.29
Parity						−0.03	0.07	−0.44	0.66	−0.17, 0.10
Single Relationship Status						0.42	0.17	2.48	0.01*	0.09, 0.76
Previous Therapy						0.21	0.24	0.85	0.46	−0.27, 0.69
*R* ^*2*^	0.10					0.14				
*Adjusted R* ^*2*^	0.09					0.10				
*F*	9.34					4.05				

*Note.* EDPS = Postpartum Depression, GRM = Gendered Racial Microaggressions, CR = Community Resilience.

Model 1 = Unadjusted Effect Modification Model, Overall Model 1 Statistics: F (3, 255) = 9.34, p < 0.001, R^2^ = 0.10 Model 2 = Adjusted Effect Modification Model, Overall Model 2 Statistics: F (10, 248) = 4.06, p < 0.001, R^2^ = 0.14

a Logarithmic transformed variable.

** *p* < 0.01

* *p* < 0.05.

**Table 7:** Unadjusted and adjusted analysis for community resilience moderating GRM from postpartum functioning.

	Postpartum Functioning (PROMIS)^a^									
	Model 1					Model 2				
	*B*	*SE*	*t*	^*p*^	95% CI	*B*	*SE*	*t*	^*p*^	95% CI
GRM^a^	−0.03	0.01	−2.40	0.02*	−0.05, −0.01	−0.03	0.01	−2.23	0.03*	−0.05, −0.00
CR^a^	0.07	0.03	2.12	0.03*	0.01, 0.14	0.08	0.03	2.24	0.03*	0.01, 0.14
GRMS X CR	−0.03	0.02	−1.43	0.15	−0.07, 0.01	−0.03	0.02	−1.24	0.16	−0.07, 0.01
Asian						0.02	0.05	0.44	0.66	−0.08, 0.13
Hispanic/Latina						−0.06	0.03	−1.73	0.09	−0.12, 0.01
Other Race						0.03	0.05	0.51	0.61	−0.07, 0.12
Delivery Mode						0.01	0.03	0.44	0.66	−0.05, 0.07
Parity						−0.01	0.01	−0.58	0.56	−0.03, 0.02
Single Relationship Status						−0.04	0.03	−1.26	0.21	−0.11, 0.23
Previous Therapy						−0.02	0.05	−0.35	0.72	−0.01, 0.07
*R* ^*2*^	0.08					0.11				
*Adjusted R* ^*2*^	0.07					0.08				
*F*	8.07					3.20				

*Note.* PROMIS = Postpartum Functioning, GRM = Gendered Racial Microaggressions, PR = Personal Resilience.

Model 1 = Unadjusted Effect Modification Model, Overall Model 1 Statistics: F (3, 255) = 8.07, p < 0.001, R^2^ = 0.08.

Model 2 = Adjusted Effect Modification Model. Overall Model 2 Statistics: F (10, 248) = 3.20, p = 0.001, R^2^ = 0.11

a Logarithmic transformed variable.

* *p* < 0.05.

**Table 8:** Unadjusted and adjusted analysis for personal resilience moderating GRM from postpartum functioning.

	*Postpartum Functioning* (PROMIS)^a^									
	Model 1					*Model 2*				
	*B*	*SE*	*t*	^*p*^	*95% CI*	*B*	*SE*	*t*	^*p*^	95% CI
GRM^a^	−0.02	0.01	−1.61	0.11	−0.04, 00	−0.02	0.01	−1.50	0.13	−0.04, 0.01
PR^a^	0.07	0.03	2.19	0.03*	0.01, 0.14	0.07	0.03	2.16	0.03*	0.01, 0.14
GRM X PR	−0.06	0.02	−2.80	0.005**	−0.09, −0.02	−0.06	0.02	−2.79	0.005**	−0.10, −0.02
Asian						0.04	0.05	0.75	0.45	−0.07, 0.15
Hispanic/Latina						−0.05	0.03	−1.40	0.16	−0.12, 0.02
Other Race						0.04	0.05	0.80	0.43	−0.06, 0.14
Delivery Mode						0.02	0.03	0.69	0.49	−0.04, 0.08
Parity						−0.01	0.01	−0.42	0.67	−0.03, 0.02
Single Relationship Status						−0.04	0.03	−1.30	0.19	−0.11, 0.02
Previous Therapy						0.00	0.05	0.04	0.97	−0.09, 0.09
*R* ^*2*^	0.10					0.12				
*Adjusted R* ^*2*^	0.09					0.09				
*F*	9.43					3.58				

*Note*. PROMIS =Postpartum Functioning, GRM =Gendered Racial Microaggressions, PR =Personal Resilience.

Model 1 = Unadjusted Effect Modification Model, Overall Model 1 Statistics: F (3, 255) = 9.43, p <0.001, R^2^ = 0.10.

Model 2 = Adjusted Effect Modification Model; Overall Model 2 Statistics: F (10, 248) = 3.58, p <0.001, R^2^ = 0.12

a Logarithmic transformed variable.

** p < 0.01

* p < 0.05.

## Footnotes

1 We use the term Global Majority to refer to Asian, Black, and Hispanic populations instead of other terms such as BIPOC, minoritized, or marginalized individuals to challenge assumptions of white normativity and to acknowledge that communities of color make up most of the global population (Maharaj and Campbell-Stephens, 2021).
